# Impact of GAN artifacts for simulating mammograms on identifying mammographically occult cancer

**DOI:** 10.1117/1.JMI.10.5.054503

**Published:** 2023-10-12

**Authors:** Juhun Lee, Tamerlan Mustafaev, Robert M. Nishikawa

**Affiliations:** a University of Pittsburgh, Department of Radiology, Pittsburgh, Pennsylvania, United States; b University of Pittsburgh, Department of Bioengineering, Pittsburgh, Pennsylvania, United States

**Keywords:** computer-aided diagnosis, occult breast cancer, conditional generative adversarial network, GAN artifacts, deep learning, artificial intelligence, image translation

## Abstract

**Purpose:**

Generative adversarial networks (GANs) can synthesize various feasible-looking images. We showed that a GAN, specifically a conditional GAN (CGAN), can simulate breast mammograms with normal, healthy appearances and can help detect mammographically-occult (MO) cancer. However, similar to other GANs, CGANs can suffer from various artifacts, e.g., checkerboard artifacts, that may impact the quality of the final synthesized image, as well as the performance of detecting MO cancer. We explored the types of GAN artifacts that exist in mammogram simulations and their effect on MO cancer detection.

**Approach:**

We first trained a CGAN using digital mammograms (FFDMs) of 1366 women with normal/healthy breasts. Then, we tested the trained CGAN on an independent MO cancer dataset with 333 women with dense breasts (97 MO cancers). We trained a convolutional neural network (CNN) on the MO cancer dataset, in which real and simulated mammograms were fused, to identify women with MO cancer. Then, a radiologist who was independent of the development of the CGAN algorithms evaluated the entire MO cancer dataset to identify and annotate artifacts in the simulated mammograms.

**Results:**

We found four artifact types, including checkerboard, breast boundary, nipple-areola complex, and black spots around calcification artifacts, with an overall incidence rate over 69% (the individual incident rate ranged from 9% to 53%) from both normal and MO cancer samples. We then evaluated their potential impact on MO cancer detection. Even though various artifacts existed in the simulated mammogram, we found that it still provided complementary information for MO cancer detection when it was combined with the real mammograms.

**Conclusions:**

We found that artifacts were pervasive in the CGAN-simulated mammograms. However, they did not negatively affect our MO cancer detection algorithm; the simulated mammograms still provided complementary information for MO cancer detection when combined with real mammograms.

## 1 Introduction

Our previous study^1^^,^^2^ showed that bilateral breast tissue difference is key to detecting mammographically occult (MO) cancer. MO cancer is a very subtle breast cancer that radiologists fail to recognize as a sign of cancer. We showed that a Radon cumulative distribution transform (RCDT)^3^ from one side of the breast to another side of the breast can amplify a very subtle bilateral breast tissue difference, which can be used to identify MO cancer.^1^^,^^2^^,^^4^^,^^5^

Conditional generative adversarial network (CGAN),^6^ precisely the pix2pix model, is a type of GAN that solves image-to-image transition problems. Image-to-image transition is a type of computer vision task that translates a given image to another in the target profile, which we can train from the given paired image dataset. A CGAN consists of a generator and a discriminator that can both access what the given image is in the CGAN setup. Thus, the generator can use the information in the given input image as a condition to create the output.

We recently tested whether a CGAN simulated mammogram can help detect MO cancer by providing additional information about the MO cancer via comparing the simulated and real images.^5^^,^^7^ Specifically, we examined whether the difference between the simulated mammogram and the real one may show the MO cancer better than comparing real breasts laterally. We used one side of the breast (e.g., left) as the condition, or template image, to guide the generation process of the opposite side of the breast (e.g., right). We trained our CGAN on left-right breast mammogram pairs of normal/healthy and asymptomatic women (BI-RADS 1); therefore, it could simulate the opposite breast mammogram with normal appearance, using the given breast as the condition/template. The simulated breast mammograms should appear as normal/healthy, so the CGAN provides additional diagnostic information about MO cancer when compared with the corresponding real mammograms. Thus, by combining the diagnostic information from real-real mammogram pairs (e.g., left-right CC view) and that from real-simulated pairs (e.g., left-simulated left CC view), our algorithm proposed in our previous study was able to identify women with MO cancer (left CC view in this example) better than the baseline model using real-real mammogram pairs only.

Although many GAN architectures have been proposed and developed for different datasets and tasks, they can still exhibit common signatures or fingerprints that can be easily identified by automated algorithms or by the human eye.^8^ These fingerprints, if they are not desirable for image synthesis or simulation, can be referred to as artifacts.

We can categorize GAN artifacts into two groups in terms of from where they originated. The first category is a network design artifact or design artifact. This is the case of a specific design of the GAN architecture creating a certain artifact. For example, the transposed convolution layer is commonly used in the generator of the GAN architectures when it is upsampling generated images. It can cause so-called checkerboard artifacts when its stride value is smaller (but greater than 2) than its kernel size.^9^ In addition to this checkerboard artifact, other common signatures that a single model could easily identify exist.^8^

The second category is an application-specific artifact for the case of the resulting GAN simulation not following the typical characteristics (or distribution) of the target images/objects. For example, if we synthesize a child’s face using a GAN, we would say a generated child’s face with a mustache and/or beard has an application-specific artifact, as it does not exhibit representative characteristics that could be sampled from the distribution of a common child’s face. Similarly, if a simulated airplane has a bent wing or body, it deviates from the common aspects of its true distribution; therefore, we know it has an application-specific artifact. For the case of simulating breast mammograms using a GAN, a non-smooth breast outline is opposite the typical characteristics of the breast shape, so it has an application-specific artifact in the context of simulating a breast mammogram.

These artifacts are common in GAN-simulated images and they certainly affect the visual quality of the simulated images (e.g., the human evaluation of how realistic the simulated images are) and the performance of the tasks of interest (e.g., image denoising).^10^ In fact, Kelkar et al.^11^ showed that several per-image statistics (e.g., signal-to-noise ratio or fat to glandular tissue ratio) of GAN-simulated images do not follow the distribution of the directly simulated images by canonical stochastic image models (SIM), such as virtual imaging clinical trials for regulatory evaluation (VICTRE) by the FDA.^12^

Hence, it is obvious that CGAN simulated mammograms will exhibit certain types of artifacts, and they can affect the MO cancer detection performance of deep models using CGAN simulated mammograms. To the best of our knowledge, there are no previous studies that have thoroughly investigated what kind of artifacts exist in GAN simulated mammograms and their possible impact on MO cancer detection. Therefore, this study evaluated CGAN simulated mammograms to discover artifacts that CGAN simulated/synthesized mammograms could exhibit and their impacts on MO cancer detection.

## 2 Methods

### 2.1 Dataset

This study used two datasets: one for developing the CGAN for simulating the opposite side of the breast mammogram and the other to test the CGAN generated mammogram for MO cancer detection. The first dataset included screening full field digital mammograms (FFDMs) of 1366 women with normal/healthy breasts (BI-RADS classification category 1). We refer to this dataset as the CGAN training dataset. Each woman in this dataset had a screening mammogram at a single time point. The second dataset included mammograms of 333 women with dense-breast tissue rated as BI-RAD breast density level 3 or level 4 (i.e., BI-RADS density level c or d). Among the 333 women, 236 were normal with two consecutive negative screening FFDMs, and 97 had unilateral MO cancer. We refer to this dataset as the MO cancer dataset. We collected both datasets under an Institutional Review Board (IRB) approved protocol. We used the most recent negative prior mammograms for the second dataset.

### 2.2 Preprocessing

We first used the existing automated algorithm^13^ to locate the breast area and to remove any unnecessary portion (e.g., view-tag and non-breast tissue). Then, we segmented the breast area in the mammogram using a tight rectangular window surrounding the breast area. We then resized each image to the size of 1024 by 1024 pixels using bicubic interpolation. We also converted the original 12-bit mammograms to 8-bit gray scale images by linearly scaling them down. We selected 1024 by 1024 pixels as the spatial resolution for developing the CGAN simulation to generate plausible mammograms while keeping the CGAN network and training images manageable within a single GPU.

### 2.3 Simulating Mammogram Using CGAN

CGAN requires input (or condition) and target image pairs for training. We used left mammograms as the input/condition and their corresponding right mammograms as the target, except for the cancer cases in the second dataset. We set the cancer side as the target and the normal contralateral side as the input. Note that we used only the first dataset (1366 negative mammograms) for training the CGAN, and the second dataset was held for testing the CGAN for detecting MO cancer.

We adopted the original CGAN setup by Isola et al.^14^ The CGAN is trained to translate the given input image x and random noise vector z to the target image y, which is formulated as G: {x,z}→y, where G indicates the generator. Generator G is trained to fool discriminator D by creating realistic fake images, whereas discriminator D is trained to detect the images by the generator as fake. The objective function of the CGAN is formulated as

$$\text{Objective}=\arg\;\underset{G}{\min}\;\underset{D}{\max}\;L_{\mathrm{cGAN}}(G,D)+\lambda L_{L1}(G),$$ (1)

where $L_{\mathrm{cGAN}}(G,D)$ and $L_{L1}(G)$ are the loss function for the CGAN and the $L_{1}$ regularization term, respectively, and are written as

$$L_{\mathrm{cGAN}}(G,D)=E_{x,y}[\log\;D(x,y)]+E_{x,z}[\log(1-D(x,G(x,z))],$$ (2)

$$L_{L1}(G)=E_{x,y,z}[{\left\|y-G(x,z)\right\|}_{1}].$$ (3)

The generator in the CGAN uses the U-net architecture as the skip connection between the encoder, and the decoder helps explore the similar characteristics that the input and the target images may have. This is also the right choice for our objective as left-right breast mammograms should share common features (e.g., breast shape and density). Note that the original CGAN built to create 256 by 256 images is too low of a resolution as the typical resolution of mammograms is 2k by 3k. We can increase the resolution of simulated mammograms by increasing the depth of the generator. However, increasing the depth to simulate 2k or 3k mammograms from a single GPU is less feasible due to the significant memory size required to hold the network, as well as process a batch of input mammograms. Thus, we increased the depth of the generator by two levels (each depth doubles the output size) to create high-resolution mammograms of 1024 by 1024 pixels without running out of memory, while still exhibiting details of mammograms.

We directly adopted the discriminator from the CGAN, called patchGAN, which focuses on the fidelity of N by N patches, instead of evaluating the entire image. The receptive field of the original patchGAN was 70 by 70 pixels.

For training a CGAN to simulate/synthesize breast mammograms, we used the Adam optimizer with a learning rate of 0.0002, and momentum parameters of $\beta_{1}=0.5$, $\beta_{2}=0.999$. In addition, we set the maximum epoch as 200, the weight for L1 regularization λ as 100, and a minibatch size as 1. We used a single Nvidia Titan X GPU for training. The training of the CGAN on our dataset took ∼48  hours.

### 2.4 MO Cancer Detection Using CGAN Simulated Mammograms

We used our RCDT and convolutional neural network (CNN) framework^1^. Briefly, we first applied the RCDT on left-right mammograms to create a single RCDT processed image; then we trained a VGG16^15^ on the RCDT processed images to identify women with MO cancer. From four standard views, i.e., left-right craniocaudal (CC) views and left-right mediolateral oblique (MLO) views, we get two RCDT images, one for the CC-view and another for the MLO-view. Using the CGAN trained on the CGAN training dataset, we generated contralateral mammograms for the MO cancer dataset using the left side (or non-cancer side) as the input. We then applied the RCDT to real-simulated pairs: (1) real right-simulated right for normal or healthy women and (2) real MO cancer side- its CGAN-simulated side with a normal or healthy appearance for MO cancer cases. This generated one additional RCDT image for the CC and MLO views. We then fused RCDT images from a real-simulated mammogram with those from real–real mammogram pairs.

**Fig. 1:**
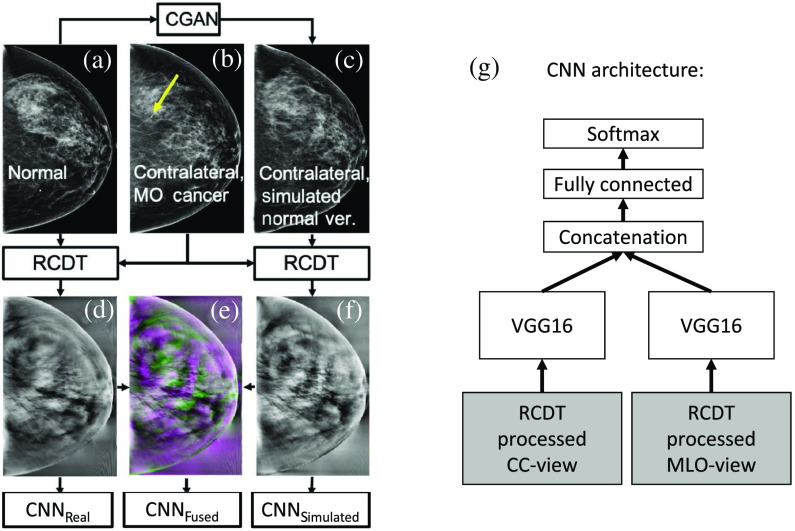
Our MO cancer detection scheme using RCDT and CNN. Using (a) a mammogram as an input for CGAN, we simulated (c) the normal version of (b) the contralateral mammogram that contains cancer. Using RCDT, we processed them to create [(d), (f)] two RCDT images and (e) fused them to highlight possible MO cancer signals, which was used to train three CNNs. We repeated this process for the MLO-view. (g) Then, each view information was fed into the deep network with two branches: one for processing the RCDT processed CC-view images and the other for processing the RCDT processed MLO-view images. We used a VGG16 without the last two layers as the backbone network.

We divided the MO cancer dataset into training, validation, and testing sets with a ratio of 7:1:2. We trained three CNNs: one trained on fused RCDT images termed ${\mathrm{CNN}}_{\text{Fused}}$, one trained only on real RCDT images termed ${\mathrm{CNN}}_{\text{Real}}$, and one trained only on simulated RCDT images termed ${\mathrm{CNN}}_{\text{Simulated}}$ (Fig. 1). Each CNN has two branches: one for processing the RCDT processed CC-view images and the other for processing the RCDT processed MLO-view images. Each branch has VGG16 without the last two layers as the backbone network. We then combined the diagnostic information from each view by passing them through a concatenation layer followed by fully-connected and softmax layers [Fig. 1(g)]. For training, we used a single Nvidia Titan X GPU with the following training parameters: Adam optimizer, a maximum epoch number of 128, mini-batch size of 16, learning rate of 0.00001, and learning rate drop factor of 0.5 with a drop period of 10. We adopted an early stopping strategy when there is no improvement during validation.

### 2.5 Evaluation

**Fig. 2:**
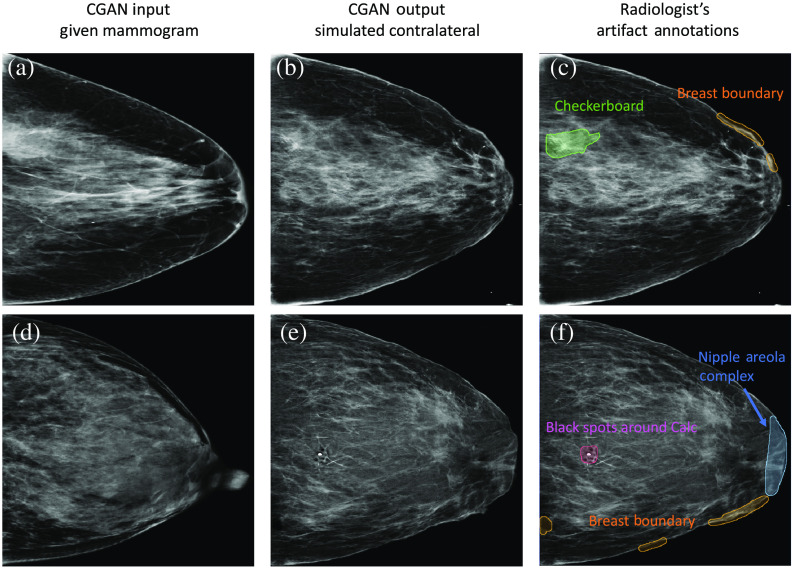
Examples of artifacts identified by the radiologist. The mammograms in the first column show the input mammograms for the CGAN simulation. The mammograms in the middle column are resulting simulated mammograms that were presented to the radiologist. The mammograms in the last column show the radiologist’s annotation with the outline overlaid on the simulated mammograms. For 236 normal controls and 97 MO cancer cases, we found one network design artifact (ND artifact) and three application-specific artifacts (AS artifact) from their corresponding simulated mammograms. They are a checkerboard artifact, a common ND artifact, which appears as repeated “X” shaped simulated dense tissue in small and fatty areas [annotated in green color in (c)]; a nipple-areola complex artifact, an AS artifact, which appears as fibroglandular tissue as seen in the middle of breast instead of showing the typical characteristics of a nipple [annotated in blue color in (f)]; a breast boundary artifact, an AS artifact, which appears as a non-smooth (or fluctuated) and/or disconnected outline of the breast [annotated in orange color in (c) and (f)]; and black spot around calcifications artifact, another AS artifact, which is not typical in mammograms [annotated in magenta color in (f)].

To investigate CGAN-generated artifacts, a radiologist (Dr. Mustafaev), who was independent of the development process of the CGAN used in this study, reviewed the CGAN-simulated mammograms of the entire MO cancer dataset to identify artifact types and their incidence rates. Dr. Mustafaev was trained and practiced radiology in Russia and is experienced in reading digital breast tomosynthesis images. If Dr. Mustafaev found an artifact that was well-defined in the literature (e.g., checkerboard artifact), he followed that definition. When he identified new artifacts that had not been reported elsewhere, he defined, categorized, and counted the new artifacts. In addition, he outlined the artifact locations in the given mammogram using an open-source segmentation algorithm called “Seg3d.”^16^ Figure 2 shows an example of artifacts with the radiologist’s outlines.

## 3 Results

### 3.1 Artifact Types and Incident Rates in CGAN Simulated Mammograms

From 236 normal controls and 97 MO cancer cases, we found one network design artifact (ND artifact), which was a checkerboard artifact, and three application-specific artifacts (AS artifact), which were nipple-areola complex, breast boundary artifacts, and black dots or spots around calcification artifacts (or black spots around Calc) from their corresponding CGAN simulated mammograms, which might impact the MO cancer detection performance of the studied CNNs.

In the simulated mammograms, the checkerboard artifacts appeared as repeated ‘X’ shaped simulated dense tissue in small and fatty areas [Fig. 2(c), annotated in green]. The nipple-areola complex artifact [Fig. 2(f), annotated in blue] appeared as fibroglandular tissue as seen in the middle of breast, instead of showing the typical characteristics of a nipple [as shown in Fig. 2(d)]. The breast boundary artifact was a non-smooth (or fluctuated) or disconnected outline of the breast [Figs. 2(c) and 2(f), annotated in orange]. The last artifact is high-contrast black spots around calcification, which is not typical in mammograms [Fig. 2(f), annotated in magenta].

Among 236 normal samples/women, we found that the simulated mammograms of 183 women (overall incident rate = 78%) showed a total of 288 artifacts, consisting of 108 cases with checkerboard artifacts (incident rate of 46%), 126 with breast boundary artifacts (incident rate of 53%), 33 with nipple-areolar complex artifacts (incident rate of 14%), and 21 with black spots around calcification artifacts (incident rate of 9%). Many simulated mammograms (90 out of 236, 38%) showed two or more artifacts in the same mammograms (mainly breast boundary and checkerboard artifacts, N=51).

Similarly, for 97 MO cancer cases, we found that the simulated mammograms of 67 women with MO cancer (overall incident rate = 69%) showed a total of 87 artifacts, consisting of 38 cases with checkerboard artifacts (incident rate of 39%), 33 with breast boundary artifacts (incident rate of 34%), 11 with nipple-areola complex artifacts (incident rate of 11%), and 16 with black spots around calcification artifacts (incident rate of 16%). Similar to the normal cases, many simulated mammograms (24 out of 97, 25%) showed two or more artifacts in the same mammograms (mainly breast boundary and checkerboard artifacts, N=10).

These results indicate that, regardless of MO cancer status, ones should expect to observe some artifacts (most likely checkerboard or breast boundary artifacts) with mammograms simulated using CGAN. In addition, two or more types of artifacts could exist in the same mammograms for one out of every three cases. Some artifacts (e.g., breast boundary artifact) could be generated in multiple locations of the same mammogram (as shown in Fig. 1). The incident rates of artifacts in the normal samples and those in the MO cancer samples are similar to each other, except for the black spot artifact. As the black spot artifact typically appears around calcifications and calcifications can be associated with breast cancer,^17^ it is logical to observe a relatively higher incident rate of black spot artifacts in the MO cancer cases than normal.

### 3.2 Analysis of Potential Impacts of Artifacts on MO Cancer Detection

**Fig. 3:**
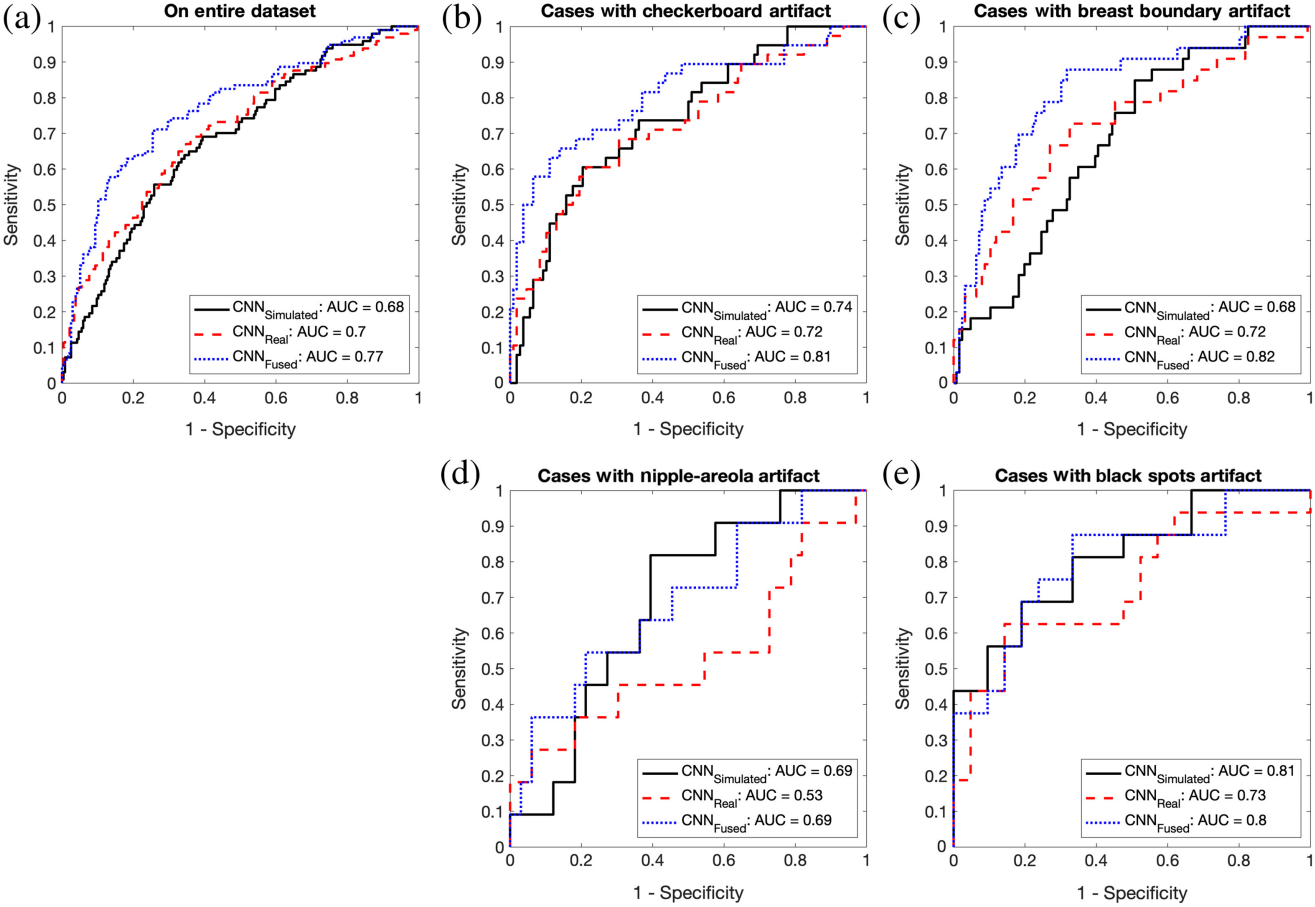
(a) The ROC curves of each model on the entire MO cancer dataset and (b)–(e) the subsets of cases with each artifact to show the overall impact of each artifact for MO cancer detection.

It is clear that CGAN simulated mammograms would exhibit some artifacts, and it is difficult to avoid them. Hence, it is now important to analyze their possible impacts on a clinical task, in this case, MO cancer detection by the CNN algorithms. The test area under the receiver operating characteristic (ROC) curves (AUC) was 0.68 with a 95% confidence interval (95% CI) of [0.62, 0.75] for ${\mathrm{CNN}}_{\text{Simulated}}$, 0.70 with a 95% CI of [0.64, 0.77] for ${\mathrm{CNN}}_{\text{Real}}$, and 0.77 with a 95% CI of [0.71, 0.83] for ${\mathrm{CNN}}_{\text{Fused}}$ for the entire MO cancer dataset [see Fig. 3(a)]. Overall, the performances of ${\mathrm{CNN}}_{\text{Simulated}}$ and ${\mathrm{CNN}}_{\text{Real}}$ are similar to each other. However, by combining diagnostic information from simulated mammograms and corresponding real mammograms, ${\mathrm{CNN}}_{\text{Fused}}$ achieved a statistically improved performance (p<0.02) over the other two CNNs, which was reported in our previous work.^5^

We then repeated the ROC analysis for the cases with each artifact to analyze its potential impacts on MO cancer detection. Figure 3 and Table 1 show the ROC curves, their associated performances (i.e., AUC), and the comparison (via bootstrap sampling) between different models for each artifact. For the checkerboard and breast boundary artifact cases, the performances of ${\mathrm{CNN}}_{\text{Simulated}}$ and ${\mathrm{CNN}}_{\text{Real}}$ are similar to each other (AUCs≈0.7), whereas ${\mathrm{CNN}}_{\text{Fused}}$ performed better than the other two models (${\mathrm{AUC}}_{\text{Fused}}\approx 0.8>{\mathrm{AUC}}_{\text{Simulated or Real}}\approx 0.7$), which follows what we found from the entire MO cancer dataset as shown in Fig. 3(a). Although checkerboard and breast boundary artifacts are pervasive in CGAN simulated mammograms, CGAN simulated mammograms still provide complementary information that real mammograms do not have, resulting in the improved MO cancer detection performance when the diagnostic information from CGAN simulated and real mammograms were combined.

**Table 1:** Impact of artifacts on MO cancer detection.

Artifact type	Model	Mean AUC [95% CI]	Mean (${\mathrm{AUC}}_{\text{Real}/\text{Fused}}$- ${\mathrm{AUC}}_{\mathrm{Simul}}$) [95% CI]	p-value
Checkerboard	${\mathrm{CNN}}_{\text{Real}}$	0.72 [0.62, 0.81]	−0.016 [−0.118, 0.086]	0.763
	${\mathrm{CNN}}_{\text{Fused}}$	0.81 [0.71, 0.89]	0.071 [−0.011, 0.154]	0.095
	${\mathrm{CNN}}_{\text{Simul}}$	0.74 [0.65, 0.82]	—	—
Breast boundary	${\mathrm{CNN}}_{\text{Real}}$	0.72 [0.61, 0.82]	0.043 [−0.052, 0.131]	0.365
	${\mathrm{CNN}}_{\text{Fused}}$	0.82 [0.73, 0.89]	0.142 [0.050, 0.235]	0.003^a^
	${\mathrm{CNN}}_{\text{Simul}}$	0.68 [0.58, 0.76]	—	—
Nipple-areola	${\mathrm{CNN}}_{\text{Real}}$	0.53 [0.31, 0.74]	−0.143 [−0.380, 0.059]	0.177
	${\mathrm{CNN}}_{\text{Fused}}$	0.69 [0.50, 0.85]	0 [−0.194, 0.187]	1
	${\mathrm{CNN}}_{\text{Simul}}$	0.69 [0.52, 0.84]	—	—
Black spots	${\mathrm{CNN}}_{\text{Real}}$	0.73 [0.55, 0.89]	−0.080 [−0.266, 0.085]	0.376
	${\mathrm{CNN}}_{\text{Fused}}$	0.8 [0.65, 0.94]	−0.012 [−0.146, 0.127]	0.906
	${\mathrm{CNN}}_{\text{Simul}}$	0.81 [0.65, 0.93]	—	—

a Statistically significant with the significant level of 0.00625 by Holm–Bonferroni correction.

For the cases with the nipple-areola complex and black spots artifacts, the performances of all CNNs were not statistically different from each other, which is mainly due to the limited sample size. However, we still observe that the performance of ${\mathrm{CNN}}_{\text{Simulated}}$ is relatively higher than that of ${\mathrm{CNN}}_{\text{Real}}$, whereas it is similar to that of ${\mathrm{CNN}}_{\text{Simulated}}$ (i.e., ${\mathrm{AUC}}_{\text{Simulated}}\approx {\mathrm{AUC}}_{\text{Fused}}>{\mathrm{AUC}}_{\text{Real}}$), which still shows the complementary aspect of CGAN simulated mammograms for MO cancer detection.

## 4 Discussion

In this study, we showed that artifacts are prevalent (in two out of every three cases) in CGAN-simulated mammograms, as previous studies have reported for different domains and tasks.^8^^–^^10^^,^^18^ From the simulated mammograms generated by our CGAN algorithm, we identified one network design artifact, which was the checkerboard artifact, and three application-specific artifacts, which were the breast boundary, nipple-areolar complex, and black spot artifacts. We found that these artifacts could appear in any mammograms regardless of the existence of MO cancer, but checkerboard and breast boundary artifacts were more common than the other two artifacts. However, we also found that mammograms with such artifacts can still provide complementary diagnostic information for MO cancer detection compared with real mammograms.

Shen et al.^9^ studied where and why checkerboard artifacts happen in medical images (vessel segmentation in retinal images). They found that checkerboard artifacts could be generated anywhere in the image, when the length of stride was smaller than the size of the convolution kernel. Checkerboard artifacts are more apparent in non-complex areas, or smooth areas, as checkerboard artifacts can stand out more easily in these area than in areas with complex textures. Specifically, they showed that checkerboard artifacts by traditional GANs (e.g., CGAN^14^) could be found in the surrounding non-target areas, e.g., surrounding background area for vessel tree segmentation in retinal images, which should be blank (completely black or white) in resulting GAN synthesized images.

Compared with the above study, we found different trends from the checkerboard artifact from our results. Unlike the study of Shen et al., our CGAN created the checkerboard artifact within the dense tissue area, which is a complex and a non-smooth portion in the breast mammogram. We rarely observed checkerboard artifacts in the fatty (darker area within the breast) and background areas (outside of the breast). However, as shown in Fig. 2(b), one can notice that the appearance of dense tissue and that of the checkerboard artifact are different. Specifically, their spatial rate of change is different; the rate of change for dense tissue is lower (i.e., smoother) than that for checkerboard artifacts. This observation may suggest that the checkerboard artifact can be visible or more identifiable as long as its spatial change rate is higher than its background. Thus, although a checkerboard artifact can happen anywhere in CGAN simulated mammograms, it may not be visible when its spatial change rate is similar to that of surrounding dense tissue.

Note that we used a CGAN to synthesize/simulate a contralateral breast (e.g., right breast) using a given breast (e.g., left breast) as a template (or input/condition) to guide the generation process. This is feasible as left and right breast tissue compositions (location and amount) of the same woman are similar to each other. A CGAN would synthesize feasible outcomes if the template images are similar to images on which the CGAN was trained. If the template image is outside the distribution of the trained images, the CGAN could create application-specific artifacts as it needs to extrapolate the given data to generate images. As shown in the template/input image in Fig. 2(d), the sample women have a prolonged nipple with high density, which is less likely seen in our CGAN training dataset. As a result, our CGAN tried to synthesize the nipple portion based on the data on which it was trained, that is, generating it as typical dense breast tissue inside the breast.

Breast boundary artifacts [e.g., Figs. 2(c) and 2(d)] could be explained similarly. When our CGAN attempted to simulate the typical shape of the breast boundary that falls outside the dataset distribution, it failed to synthesize feasible outcomes. Specifically, we observed that the breast boundary artifact may be related to how close the dense tissue is to the breast boundary. As shown in Fig. 2(b), the breast outline of simulated mammograms displayed a smooth profile when there was no dense tissue nearby. However, when dense tissue was present around the nipple area, the breast outline in that region appeared to be pulled toward the dense tissue. As breast density increases, more dense tissue may appear near the breast boundary, potentially leading to an increased occurrence of breast boundary artifacts. Therefore, it is possible that extremely dense breasts would exhibit more breast boundary artifacts than others. As gathering cases with extremely dense breasts (∼10% of the population) can be challenging, the CGAN may struggle to learn better representations of denser breasts.

For the case of black spot artifacts, they tend to appear more frequently in MO cancer cases (Sec. 3.1). As calcifications can be associated with breast cancer, the input or condition mammogram used for CGAN simulation in MO cancer cases may have more calcifications than in normal controls. Consequently, our CGAN may be forced to add calcifications to the simulated mammograms for MO cancer cases. This choice of adding calcifications may be a safer option for the algorithm to reduce the loss (i.e., the difference between the simulated and target mammograms) because the target mammograms from the dataset might have a higher frequency of calcifications. However, not all cancer cases are associated with calcifications, and including more cases without calcifications could help reduce the frequency of black spot artifacts. In summary, the limited diversity in the training dataset could be the main reason for the occurrence of application-specific artifacts.

There are a couple of limitations in our study. First, we based our CGAN on the original pix2pix architecture by Isola et al.^6^ with a few modifications to simulate large mammograms with 1024 by 1024 pixels. Since its first appearance, various advanced GANs specifically designed for medical images have been introduced (to name a few, Refs. 9, 10, 18). Among those, Shen et al.^9^ proposed a deformable GAN, which employs deformable convolution and region of interest (ROI) pooling operations into the GAN architecture to remove checkerboard artifacts. Briefly, deformable convolution and ROI pooling were originally introduced by Dai et al.^19^ Unlike original convolution and pooling, which uses a fixed geometric sampling grid (e.g., 3×3), deformable convolution augments spatial sampling locations for convolution and pooling operations. Shen et al. used their proposed deformable GAN to synthesize various medical images and tasks (e.g., translating PET image → CT image and vessel tree image → retinal image) with fewer checkerboard artifacts than a regular GAN. Thus, we will employ a deformable GAN into our framework as a future study to check if this could remove checkerboard artifacts and improve the MO cancer detection performance.

The second limitation of our study is the limited number of samples that we used for training the CGAN for simulating mammograms. Although we used breast mammograms of 1366 normal/healthy women to train our CGAN, it cannot cover the true distribution of women’s breasts. The existence of application-specific artifacts is key evidence of the weakness of our CGAN on generality. The number of publicly available breast mammogram datasets from various vendors, geological cites, and racial backgrounds is increasing (e.g., Refs. 20 to 22). In our future work, we will include such new datasets to reduce application-specific artifacts and ultimately to increase the generality of our CGAN model to generate more realistic mammograms.

Another limitation of our study is the generalizability of our findings on the impacts of CGAN artifacts on MO cancer detection as our detection scheme is one of many solutions for MO cancer detection. It is possible that the complementary aspect of CGAN simulated mammograms is only applicable to our MO cancer detection scheme. Mainprize et al. (e.g., Ref. 23) investigated the masking effect of dense tissue for breast cancer, and CGAN artifacts could potentially have a negative impact on their framework. Thus, we will analyze the potential impacts of CGAN simulated artifacts on other MO cancer related works as a future study.

Note that our CGAN can simulate a mammogram using its contralateral mammogram as condition images. Therefore, it is obvious that the breast tissue profiles in the input/condition mammograms can affect the simulation result. As a future study, we intend to investigate the potential relationship between GAN artifacts and the features summarizing breast tissue characteristics. In fact, Kelka et al. used radiomic features, such as gray-level co-occurrence matrices, to evaluate the GAN simulated lumpy background over the direct and mathematical simulation using lump background model^24^ and found the difference in the radiomic feature distribution between GAN simulated and mathematically simulated images.^11^ Thus, we will use such radiomic features on our dataset (i.e., input mammograms for our CGAN model) as well as patient variables, such as age, breast density, and body mass index, to identify specific subpopulations that may be more susceptible to the GAN artifacts that we reported in this study. For this specific analysis, we may use virtual clinical trial software, such as VICTRE,^25^ to systematically evaluate the relationship between the GAN artifacts and various patient clinical variables as it can provide a controlled environment for breast shape, size, and density.

Moreover, different initialization of our CGAN can affect the resulting simulated mammograms as well as artifacts on them. It is possible that some artifacts could appear consistently regardless of initialization but some could vary over trials. As a future study, we will conduct how different random initializations on our CGAN would impact the resulting artifacts, whether they appear the same location or same type of artifacts appears in the mammograms.

In conclusion, we showed that artifacts are very common in CGAN simulated mammograms. However, they still have complementary information for MO cancer detection when combined with real mammograms. Further studies are necessary to find ways to reduce the artifacts that we observed, identify subpopulations that may exhibit more or fewer artifacts, and investigate the potential impacts of artifacts on other MO cancer detection frameworks.

## Code, Data, and Materials Availability

The data that support the findings of this article are not publicly available due to privacy concerns. They can be requested from the corresponding author at the email listed above.
